# Computed Tomography and Spirometry Can Predict Unresectability in Malignant Pleural Mesothelioma

**DOI:** 10.3390/jcm10194407

**Published:** 2021-09-26

**Authors:** Alice Bellini, Andrea Dell’Amore, Chiara Giraudo, Antonella Modugno, Nicol Bernardinello, Stefano Terzi, Giovanni Zambello, Giulia Pasello, Andrea Zuin, Federico Rea

**Affiliations:** 1 Thoracic Surgery Unit, Thoracic Surgery Division, Department of Cardiac, Thoracic, Vascular Sciences and Public Health, University of Padova, 35128 Padova, Italy; alicebellini26@gmail.com (A.B.); ste.terzi@gmail.com (S.T.); giovanni.zambello@aopd.veneto.it (G.Z.); andrea.zuin@unipd.it (A.Z.); federico.rea@unipd.it (F.R.); 2 Department of Medicine—DIMED, University of Padova, 35128 Padova, Italy; chiara.giraudo@unipd.it (C.G.); modugno.a@libero.it (A.M.); 3 Respiratory Disease Unit, Respiratory Division, Department of Cardiac, Thoracic, Vascular Sciences and Public Health, University of Padova, 35128 Padova, Italy; nicol.bernardinello@unipd.it; 4 Medical Oncology, Veneto Institute of Oncology IOV IRCCS, 35128 Padova, Italy; giulia.pasello@iov.veneto.it

**Keywords:** mesothelioma, thoracic surgery, RECIST

## Abstract

Preoperative identification of unresectable pleural mesothelioma could spare unnecessary surgical intervention and accelerate the initiation of medical treatments. The aim of this study is to determine predictors of unresectability, testing our impression that the contraction of the ipsilateral hemithorax is often associated with exploratory thoracotomy. Between 1994 and 2020, 291 patients undergoing intended macroscopic complete resection for mesothelioma after chemotherapy were retrospectively investigated. Eligible patients (*n* = 58) presented a preoperative 3 mm slice-thickness chest computed tomography without pleural effusion or hydropneumothorax. Lung volumes (segmented using a semi-automated method), modified-Response Evaluation Criteria in Solid Tumors (RECIST) measurements, and spirometries were collected after chemotherapy. Multivariable analysis was performed to determine the predictors of unresectability. An unresectable disease was found at the time of operation in 25.9% cases. By multivariable analysis, the total lung capacity (*p* = 0.03) and the disease burden (*p* = 0.02) were found to be predictors of unresectability; cut-off values were <77.5% and >120.5 mm, respectively. Lung volumes were not confirmed to be associated with unresectability at multivariable analysis, probably due to the correlation with the disease burden (*p* < 0.001; r = −0.4). Our study suggests that disease burden and total lung capacity could predict MPM unresectability, helping surgeons in recommending surgery or not in a multimodality setting.

## 1. Introduction

Malignant pleural mesothelioma (MPM) is an aggressive asbestos-related tumor with a poor prognosis. To date, multimodality treatment including chemotherapy and surgery, with or without radiotherapy, is the gold standard therapy for selected patients with epithelial and early stage MPM [1]. In this setting, the goal of surgery is to achieve the macroscopic complete resection (MCR) [2], obtained by either extrapleural pneumonectomy (EPP) or pleurectomy/decortication (PD). The average rate of MCR reported in the literature is 70% [3]; thus, 30% of patients underwent aborted resection due to a disease technically unresectable found at the time of surgery. The preoperative identification of an unresectable MPM could avoid futile explorative thoracotomy (ET) with R2 resection, accelerate the initiation of medical therapies, and prevent unnecessary costs to the National Health System.

In our experience, the most common factor precluding MCR is the diffuse chest wall invasion (DCWI), frequently associated with the contraction of the ipsilateral hemithorax, that has higher pleural thickness and lower aerated lung volumes. The aim of this study is to determine preoperative predictors of unresectability, testing our anecdotal impression that the contraction of the ipsilateral hemithorax is often associated with ET.

## 2. Material and Methods

### 2.1. Patient Selection

**Figure 1:**
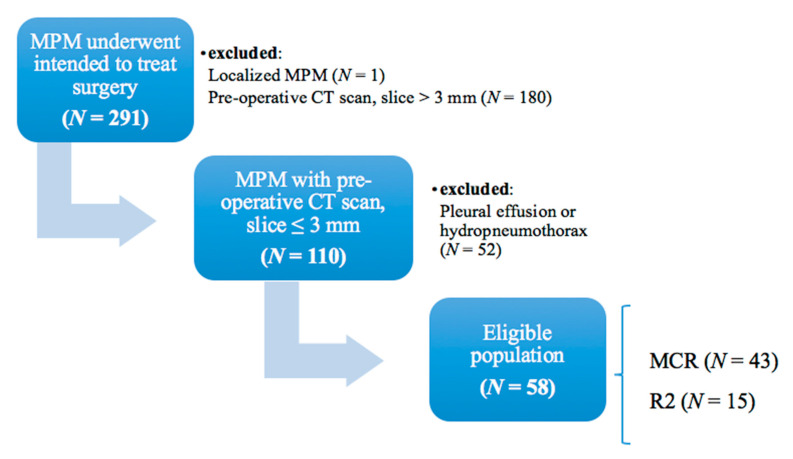
Flow diagram detailing study cohort. Final eligible population was composed by 43 patients underwent macroscopic complete resection (MCR), and 15 patients with an unresectable disease underwent explorative thoracotomy with a R2 resection (R2). MPM: malignant pleural mesothelioma; CT: computed tomography.

Between July 1994 and August 2020, 291 patients undergoing intended MCR for MPM after iCT at Padova University Hospital were retrospectively investigated. The data collection and study protocol have both been approved by the local ethics committee (n.pd732-2220T), and participating patients were asked to sign a written consent. Eligible patients (*n* = 58) were those with a preoperative 3 mm slice-thickness chest computed tomography (CT) scan without pleural effusion or hydropneumothorax (Figure 1).

Demographics (age at surgery and sex) and all relevant clinical and radiological variables, i.e., histology, side, talc pleurodesis, pathological stage, preoperative pulmonary function tests (PFTs), preoperative lung ventilation/perfusion scan, standardized uptake volume (SUV) max and metabolic response at post-iCT 18F-fluorodeoxyglucose positron emission tomography/CT (18F-FDG PET/CT) scan, lung volume measurements, and pleural thickness (disease burden and maximum pleural thickness at each level) according to Response Evaluation Criteria in Solid Tumors (RECIST) modified criteria, were collected in order to identify possible predictors of unresectability.

### 2.2. Preoperative Evaluation and Surgical Approach

At our institution, eligibility criteria for multimodality treatment included biopsy-proven MPM (of any histological subtype) at clinical stage T1-3 N0-1 M0 and anticipated complete resectability by EPP or PD, as estimated by an experienced thoracic surgeon in a multidisciplinary setting.

We prefer to perform chemotherapy in preparation for surgery for hopeful MCR. In fact, according to our experience, induction chemotherapy (iCT) (a) can be administered with high dosage in patients no longer debilitated by surgery; (b) can lead to a down-staging of the disease, allowing obtaining a satisfactory MCR; and (c) allows for a better surgical selection based on the response to chemotherapy (a poor response may avoid an unnecessary surgical treatment in a more aggressive disease). For the iCT, a platinum-based regimen with gemcitabine or pemetrexed was used for two to six cycles. Subsequently, patients were preoperatively evaluated with a chest CT scan, a 18F-FDG PET/CT scan (since 2010), an echocardiogram, PFTs, cardiopulmonary exercise testing, and a lung ventilation/perfusion scan.

In particular, the percentage to predicted levels of current volume (CV), forced vital capacity (FVC), forced expiratory volume in 1s (FEV1), total lung capacity (TLC), and diffusion lung capacity for carbon monoxide (DLCO) were collected with a spirometer (Biomedin, Padova, Italy) using a standardized method [4,5,6]. Interpretation of the spirometric data was performed following European Respiratory Society/American Thoracic Society guidelines [7].

Surgery was performed within 4–6 weeks of completing the final cycle of chemotherapy in patients whose pathology had at least been stabilized at the CT scan and PET/CT scan.

The eighth edition of the lung cancer tumor, node, and metastasis (TNM) staging system was used to define the extent of the disease [8]. The PD was based on total visceral and parietal pleura removal, while the EPP was employed in case of macroscopic pulmonary parenchyma invasion, both through an extended posterolateral thoracotomy (including sixth rib resection and/or multilevel thoracotomies). The resection and reconstruction of the pericardium and/or diaphragm were performed only in the case of macroscopic involvement. MCR was defined as the removal of all grossly visible and palpable tumors. ET for unresectable MPM, secondary either to DCWI or to the invasion of intrathoracic organs, was labeled as R2 resection. DCWI was defined as an intraoperative finding of tumor invasion through the endothoracic fascia into more than two intercostal muscles or ribs and/or multifocal invasion of the chest wall precluding MCR [3].

### 2.3. Radiological Evaluation

Two thoracic radiologists measured aerated lung volumes and pleural thickness using an Open Source Software (3D Slicer, Brigham and Women’s Hospital, Harvard University, NIH, www.slicer.org; downloaded on 11 June 2020) using contrast-enhanced CT images with 3 mm slice thickness. Pulmonary segmentation was performed via a semi-automated method [9]. First, a gray level threshold between −1020 and −250 Hounsfield units was applied. Subsequently, the airways (main bronchi and trachea) were cropped out and the volume (cm^3^) of each lung separately computed. The volumetric difference between the lung affected by MPM and the contralateral one was calculated.

The pleural thickness was evaluated according to RECIST modified criteria [10], subdividing the hemithorax divided into three levels: the upper level extended from the apex of the lung to the inferior margin of the aortic arch, the middle level included the pleura between the upper and lower levels, and the lower level below the left atrium. In the case of lesions too small to measure and in the case of the absence of lesions, default values of 5 and 0 mm were assigned, respectively. The two maximum tumor thicknesses perpendicular to the chest wall or mediastinum were measured at each level, and the sum of the six measurements was reported as the disease burden (mm).

### 2.4. Statistical Analysis

The data were reported as absolute numbers, percentages, or median values with interquartile range (IQR). The Fisher exact test and the Mann–Whitney test were performed to analyze categorical and continuous variables, respectively. The Spearman test was employed to evaluate any correlation between variables. Optimal cut-off values were determined by maximizing the sum of sensitivity and specificity. Variables with a *p*-value < 0.05 at univariable regression analysis were entered into multivariable analysis in order to evaluate independent predictors of unresectability. A *p*-value < 0.05 was considered statistically significant. All tests were two-tailed. All the statistical analyses were performed using SPSS 27 version for windows (SPSS, Inc., Chicago, IL, USA) and GraphPad Prism 8 Version 8.4.3 for macOS.

## 3. Results

### 3.1. Patient Characteristics

Patient characteristics are summarized in Table 1.

Forty-three patients attained MCR, 33 (77%) with PD and 10 (23%) with EPP, while 15 patients were found to be R2 resections, due to DCWI (*n* = 13), aortic adventitia infiltration (*n* = 1), and diaphragmatic pillar infiltration (*n* = 1). The two groups (MCR and R2) were homogeneous for age, side, iCT (regimen and number of cycles administered), SUV max, and metabolic response at 18F-FDG PET/CT scan after iCT, talc pleurodesis, histology (epithelial vs non-epithelial), and clinical stage. Conversely, the R2 group included more males (*p* = 0.01), lower PFTs (VC% *p* < 0.001; FVC% *p* < 0.001; FEV1% *p* = 0.007; TLC% *p* = 0.003; DLCO *p* = 0.04), lower ipsilateral pathological lung volume (*p* = 0.03), higher volumetric difference between the contralateral and the ipsilateral pathological lung (*p* = 0.01), and higher disease burden (*p* = 0.001). Moreover, the R2 group presented a trend toward lower ventilation (*p* = 0.07) and perfusion (*p* = 0.08) measurements at the preoperative ventilation/perfusion scan. Clinical and radiological characteristics of the 15 patients who underwent ET are reported in Table 2.

### 3.2. Preoperative Predictors of Unresectability in MPM

Table 3 displays the results of univariable and multivariable regression analysis performed to determine the association of preoperative factors with unresectability.

In univariable analysis, the TLC% (*p* = 0.005), the ipsilateral pathological lung volume (*p* = 0.04), the volumetric difference between the contralateral and the ipsilateral pathological lung (*p* = 0.03), and the disease burden (*p* = 0.002) were associated with ET, while only the TLC% (*p* = 0.03, OR 0.920 95%CI 0.853–0.992) and the disease burden (*p* = 0.02, OR 1.020 95%IC 1.003–1.038) were confirmed as independent predictors of unresectability in multivariable analysis. The optimal cut-off value for the TLC% and the disease burden as predictors of unresectability were <77.5% (AUC = 0.81, *p* < 0.001, sensitivity = 73.3%, specificity = 80%) and >120.5 mm (AUC = 0.77, *p* = 0.002, sensitivity = 60%, specificity = 90.7%), respectively.

**Figure 2:**
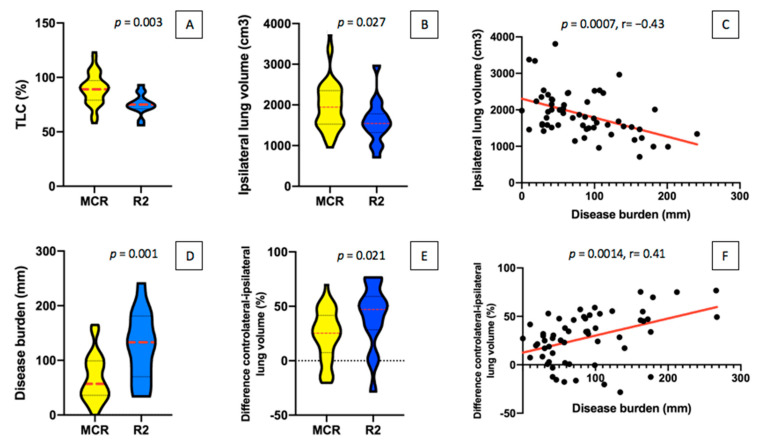
Comparison of between resectable (MCR) and unresectable (R2) cohorts in TLC% (**A**), ipsilateral lung volume (**B**), disease burden (**D**), and difference in contralateral and ipsilateral lung volume (**E**). Correlation between the increase of disease burden with both the decrease of the ipsilateral pathological lung volume (**C**) and the increase of the difference between the contralateral and the ipsilateral pathological lung (**F**). TLC: total lung capacity.

**Figure 3:**
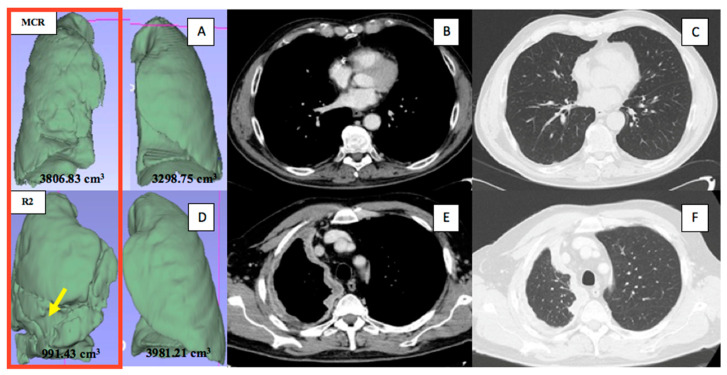
Representative radiological measurements. Upper panels, a patient with a right-sided MPM affected by the lowest disease burden, who underwent macroscopic complete resection: the aerated ipsilateral and contralateral lung volumes (**A**) and the pleural thickness (too small to measure) in the axial CT images on the portal venous phase (**B**) and the lung window (**C**), at the medium level. Lower panels, a patient with a right-sided MPM affected by the highest disease burden (the arrow indicated the furrows on the three-dimensional lung reconstruction caused by the pleural disease), who underwent R2 resection: the aerated ipsilateral and contralateral lung volumes (**D**) and the pleural thickness in the axial CT images on the portal venous phase (**E**) and the lung window (**F**), at the upper level.

Linear regression analysis demonstrated a correlation between the variation of the disease burden and the variation in ipsilateral pathological lung volume (*p* < 0.001; r = −0.4) and in the difference between the contralateral and the ipsilateral pathological lung (*p* = 0.001; r = 0.4). The optimal cut-off value for the ipsilateral pathological lung volume and the volumetric difference between the contralateral and the ipsilateral pathological lung were <1794 cm^3^ (AUC = 0.69, *p* = 0.03, sensitivity = 80%, specificity = 58.2%) and >1298 cm^3^ (AUC = 0.70, *p* = 0.02, sensitivity = 60%, specificity = 81.4%) or >46.22% (AUC = 0.71, *p* = 0.02; sensitivity = 60%, specificity = 81.4%), respectively (Figure 2 and Figure 3).

In the MCR group, 36 (84%) and 39 (91%) patients presented the TLC ≥ 77.5% and the disease burden ≤ 120.5 mm, respectively, whereas 34 (79%) had both. In the R2 group, 11 (73%) and 9 (60%) patients had the TLC < 77.5% and the disease burden > 120.5 mm, respectively, while 5 (33%) presented both.

## 4. Discussion

### 4.1. MCR Is the Central Principle of Surgery for MPM

The MPM represents a challenge for surgeons in defining the oncological principles of resection, due to its peculiar growth along the pleural surface with the predilection for local invasion. Compared to most solid tumors in which the anatomical resection can provide a microscopic free margin (R0 resection), often avoiding direct manipulation of the tumor itself, in MPM this is technically very difficult to obtain; thus, the optimal result of most surgical interventions is an MCR with microscopic positive margins (R1 resection) [3,11,12,13].

Hence, MCR has become the central principle of surgery for MPM, supported by retrospective evidence highlighting advantages in survival when compared to R2 resection [3,14,15,16]. According to a recent literature review, 30% of patients underwent aborted resection due to a disease technically found unresectable at the time of surgery [3]. Therefore, improving the preoperative identification of unresectable MPM could avoid futile ET, accelerate the initiation of medical therapies, and prevent unnecessary costs to the National Health System [17]. In our experience, the most common factor precluding MCR is DCWI, which is frequently associated with the contraction of the ipsilateral hemithorax.

### 4.2. The Role of CT and Spirometry

Consequently, we analyzed the radiological parameters that correlated with the contracted hemithorax (that is aerated lung volumes and pleural thickness) and PFTs (particularly TLC% as an indicator of restrictive syndrome) as possible preoperative predictors of unresectability. Recently, Burt and collaborators created a novel three-dimensional radiographic metric of the thoracic cage volume (TCV) and demonstrated that a 5% decrease in TCV compared with the contralateral side was significantly associated with unresectability due to DCWI [3]. Nevertheless, the aforementioned novel method was based on a fully manual segmentation and, when we tried to reproduce the same measurements, it required almost two hours per patient. For this reason, in order to test our hypothesis, we used two methods already codified in the literature, which are faster and easier to reproduce: the semi-automated segmentation of the aerated lung volumes (thirty minutes per patient) [9] and the RECIST modified criteria measuring pleural thickness (ten minutes per patient) [10]. Both lung volumes and pleural thickness according to RECIST modified criteria play an important and consolidated prognostic role in MPM survival [9,10,18,19,20,21], but to the best of our knowledge, they have not ever been tested as predictors of unresectability. The TLC% (*p* = 0.03, OR 0.920 95%CI 0.853–0.992) and the disease burden (*p* = 0.02, OR 1.020 95%IC 1.003–1.038) were found to be independent predictors of unresectability in multivariable analysis, with an optimal cut-off value of <77.5% (AUC = 0.81, *p* < 0.001, sensitivity = 73.3%, specificity = 80%) and >120.5 mm (AUC = 0.77, *p* = 0.002, sensitivity = 60%, specificity = 90.7%), respectively; whereas aerated lung volumes were significantly associated with ET only in univariable analysis, probably due to the strong correlation with the disease burden. In fact, the linear regression analysis highlighted a correlation between the increase of disease burden with both the decrease of the ipsilateral pathological lung volume (*p* < 0.001; r = −0.4) and the increase of the difference between the contralateral and the ipsilateral pathological lung (*p* = 0.001; r = 0.4). Over time, MPM inevitably leads to a restrictive syndrome (TLC < 81%); in fact, the pleural thickness squeezes the lung parenchyma and makes it become stiffer. Moreover, it reduces thoracic cage expansion and diaphragmatic mobility, causing an impairment to respiratory mechanics with ventilator pump failure [22,23]. We previously reported the role of PFTs as indicators of cytoreductive efficacy of iCT [22,23]. With this study, we add an important role to PFTs: they could be an additional tool to better improve the preoperative identification of MPM disease not amenable to MCR, mostly if a higher disease burden is present. Pleural thickness has been recently reported as a useful prognostic indicator of MPM: the International Association for the Study of Lung Cancer recently revised the definition of MPM staging (Eighth Edition) and mentioned that pleural thickness might be useful in T-component evaluation [20,21,24]. The two patients in our study with aortic adventitia infiltration and diaphragmatic pillar infiltration presented disease burden values of 183 and 134 mm, respectively; both were identified at the cut-off level. Therefore, compared to Burt et al. who considered only unresectability due to DCWI [3], the use of the disease burden according to RECIST modified criteria also could individuate the unresectable disease secondary to the invasion of the intrathoracic organs.

### 4.3. Limitations of the Study

This study presents some limitations including, firstly, its retrospective nature. Secondly, PTFs results are strongly dependent on the compliance of the patient and could be impaired in the event of inadequate pain control. Thirdly, most patients received talc pleurodesis: we were unable to evaluate the influence of this procedure on PFTs and on radiological assessment. Fourthly, it should be addressed that we have excluded patients with pleural effusion or hydropneumothorax because the effusion may have hampered the measurement of pleural thickness and, given that the first step of the applied semi-automatic segmentation of the lungs was based on a threshold, the air component of the hydropneumothorax may have caused a bias in the computation of pulmonary volumes. Lastly, we acknowledge that the magnetic resonance imaging (MRI) is superior to CT for prediction of the chest wall invasion, but we did not perform it routinely in the pre-operative evaluation, representing a limitation of our study.

## 5. Conclusions

Our study suggests that disease burden, in addition to the prognostic role already known in the literature, and TLC% could predict MPM unresectability. Simple, non-invasive, and inexpensive tests (computed tomography and spirometry) can help surgeons in recommending surgery or not in a multimodality setting. This data should be validated further by prospective studies with larger samples.

## Figures and Tables

**Table 1:** Patient characteristics.

Characteristics	MCR, *N* = 43	R2, *N* = 15	*p*-Value
Age (years), median (IQR)	64 (56–68)	63 (59–69)	0.6
Sex, *n* (%)			0.01
Male	25 (58)	14 (93)	
Female	18 (42)	1 (7)	
Side, *n* (%)			0.4
Right	22 (51)	10 (67)	
Left	21 (49)	5 (33)	
iCT regimen, *n* (%)	Platinum + pemetrexed, 42 (97.7) gemcitabine, 1 (2.3)	Platinum + pemetrexed, 13 (87) Platinum + gemcitabine, 2 (13)	0.2
iCT cycles, median (IQR)	4 (4–5.25)	4 (4–4)	0.5
CV (%), median (IQR)	92.5 (78–106.8)	73 (65–77)	<0.001
FVC (%), median (IQR)	92 (78–105.3)	73 (60–75)	<0.001
FEV1 (%), median (IQR)	89 (77–102)	77 (67–87)	0.007
TLC (%), median (IQR)	89 (79–97)	75 (71–80)	0.003
DLCO (%), median (IQR)	76 (63–87)	66 (51–77)	0.04
Scintigraphy scan, *n* (%)			0.3
No	10 (23)	6 (40)	
Ventilation/Perfusion	30 (70)	9 (60)	
Perfusion only	3 (7)	0 (0)	
Ipsilateral lung perfusion (%), median (IQR)	37.76 (32.57–45.88)	34.05 (19.62–38.86)	0.08
Ipsilateral lung ventilation (%), median (IQR)	33.31 (25–44.88)	28.4 (9.92–35.34)	0.07
Post-induction PET/CT, *n* (%)			0.2
No	11 (26)	3 (20)	
Negative/reduced	16 (37)	3 (20)	
Stable/augmented	16 (37)	9 (60)	
Preoperative SUV max, median (IQR)	7.58 (2.53–11.23)	7.95 (5.99–11.52)	0.3
Talc pleurodesis, *n* (%)			0.1
No	12 (28)	8 (53)	
Yes	31 (72)	7 (47)	
Histology, *n* (%)			>0.99
Epithelial	37 (86)	13 (87)	
Non-epithelial	6 (14)	2 (13)	
cTNM8, *n* (%)			>0.99
I	34 (79)	12 (80)	
II	9 (21)	3 (20)	
pTNM8, *n* (%)			<0.001
Complete remission-I–II	29 (67)	0 (0)	
III–IV	14 (33)	15 (100%)	
Ipsilateral pathological lung volume (cm^3^), median (IQR)	1944 (1528–2352)	1545 (1322–1782)	0.03
Difference in contralateral and ipsilateral lung volume (cm^3^), median (IQR)	677.2 (217.4–1252)	1371 (667.4–2164)	0.02
Difference in contralateral and ipsilateral lung volume (%), median (IQR)	25.28 (7.4–41.73)	47.01 (28.39–59.01)	0.01
Max pleural thickness at upper level (mm), median (IQR)	10 (5–20)	23 (9–32)	0.002
Max pleural thickness at medium level (mm), median (IQR)	12 (5–21)	22 (13–35)	0.007
Max pleural thickness at inferior level (mm), median (IQR)	13 (5–21)	28 (17–43)	0.005
Disease burden (mm), median (IQR)	57 (36–99)	133 (70–181)	0.001

MCR = macroscopic complete resection; iCT = induction chemotherapy; CV = current volume; FVC = forced vital capacity; FEV1 = forced expiratory volume in 1 s; TLC = total lung capacity; DLCO = diffusion lung capacity for carbon monoxide; PET/CT = positron emission tomography/computed tomography; cTNM8 = clinical tumor, node and metastasis Eight Edition; pTNM8 = pathological tumor, node and metastasis Eight Edition.

**Table 2:** Characteristic of 15 patients who underwent exploratory thoracotomy.

Patient	Sex	Side	Cause of R2	TLC (%)	Disease Burden (mm)	Ipsilateral Lung Volume (cm^3^)	Difference Contralateral-Ipsilateral Lung Volume (%)
1	M	Right	DCWI	88	241	1337.17	49.25
2	M	Left	DCWI	71	201	989.9	76.68
3	M	Right	DCWI	75	99	1512.07	59.01
4	M	Right	DCWI	73	58	2130.43	1.88
5	M	Right	DCWI	77	35	1584.56	52.92
6	M	Left	Aortic adventitia infiltration	86	183	2009.94	33.98
7	M	Left	DCWI	93	133	1683.51	28.39
8	M	Right	DCWI	72	181	991.43	75.10
9	M	Right	DCWI	74	70	1776.78	46.35
10	M	Right	Diaphragmatic pillar infiltration	71	134	2964.71	−28.35
11	M	Right	DCWI	80	140	1545.46	47.01
12	M	Left	DCWI	56	162	711.33	75.26
13	M	Right	DCWI	75	89	1471.99	34.46
14	M	Left	DCWI	60	123	1321.67	55.41
15	F	Right	DCWI	77	34	1782.33	0.74

TLC = total lung capacity; M = male; F = female; DCWI = diffuse chest wall invasion.

**Table 3:** Univariable and multivariable regression analysis.

	Univariable	Multivariable	
	*p*-Value	OR (95%CI)	*p*-Value
TLC (%)	0.005	0.920 (0.853–0.992)	0.03
Ipsilateral pathological lung volume (cm^3^)	0.04	1.000 (0.998–1.002)	0.9
Difference in contralateral and ipsilateral lung volume (%)	0.03	0.995 (0.955–1.037)	0.8
Disease burden (mm)	0.002	1.020 (1.003–1.038)	0.02

TLC = total lung capacity.

## Data Availability

The data presented in this study are available on request from the corresponding author.
